# Gregor Mendel’s legacy in quantitative genetics

**DOI:** 10.1371/journal.pbio.3001692

**Published:** 2022-07-19

**Authors:** Trudy F. C. Mackay, Robert R. H. Anholt

**Affiliations:** Center for Human Genetics and Department of Genetics and Biochemistry, Clemson University, Greenwood, South Carolina, United States of America

## Abstract

Mendel’s discovery of the laws of segregation and independent assortment and inference of the existence of non-Mendelian interactions between loci are at the heart of modern explorations of the genetic architecture of quantitative traits.

## Teaser

Gregor Mendel’s discovery of the laws of segregation and independent assortment and his inference of the existence of non-mendelian interactions between loci remain at the heart of today’s explorations of the genetic architecture of quantitative traits.

The field of genetics was born with the publication in 1866 of Gregor Mendel’s Experiments in Plant Hybridization [1]. Working with the garden pea, *Pisum sativum*, Mendel chose 7 “characters” (polymorphic loci each affecting a different phenotype in today’s parlance) that “permit of a sharp and certain separation,” excluding those for which “the difference is of a ‘more or less’ nature, which is often difficult to define” [1]. Mendel chose these loci because the hybrid (F_1_) between the homozygous parental genotypes (to use modern terminology) was indistinguishable from one of the parents; i.e., one of the alleles was dominant and the other recessive (Mendel’s original terms). He excluded phenotypes that were intermediate between the 2 parents in the F_1_ hybrid.

When Mendel crossed each of the 7 F_1_ hybrids among themselves, he observed a 3:1 ratio of dominant to recessive phenotypes in the F_2_ generation. He inferred that the F_1_ hybrids have 2 alleles at each locus (A, a), which are present in equal proportions in the female and male gametes, and combine at random to produce F_2_ offspring in the ratio 1 AA:2 Aa:1 aa (the Law of Segregation). Mendel also performed crosses in which the hybrids differed for 2 or 3 of the 7 loci, analyses of which led him to conclude “the relation of each pair of different characters in hybrid union is independent of the other differences in the 2 original parental stocks” (the Law of Independent Assortment) [1]. Luckily for Mendel, the 7 loci were each on a different autosome. In a separate series of crosses between 2 species of common bean with different flower colors and unexpected ratios of flower color in hybrids, Mendel correctly inferred multiple loci with recessive epistasis (where the expression of one gene is modified by another).

**Fig 1:**
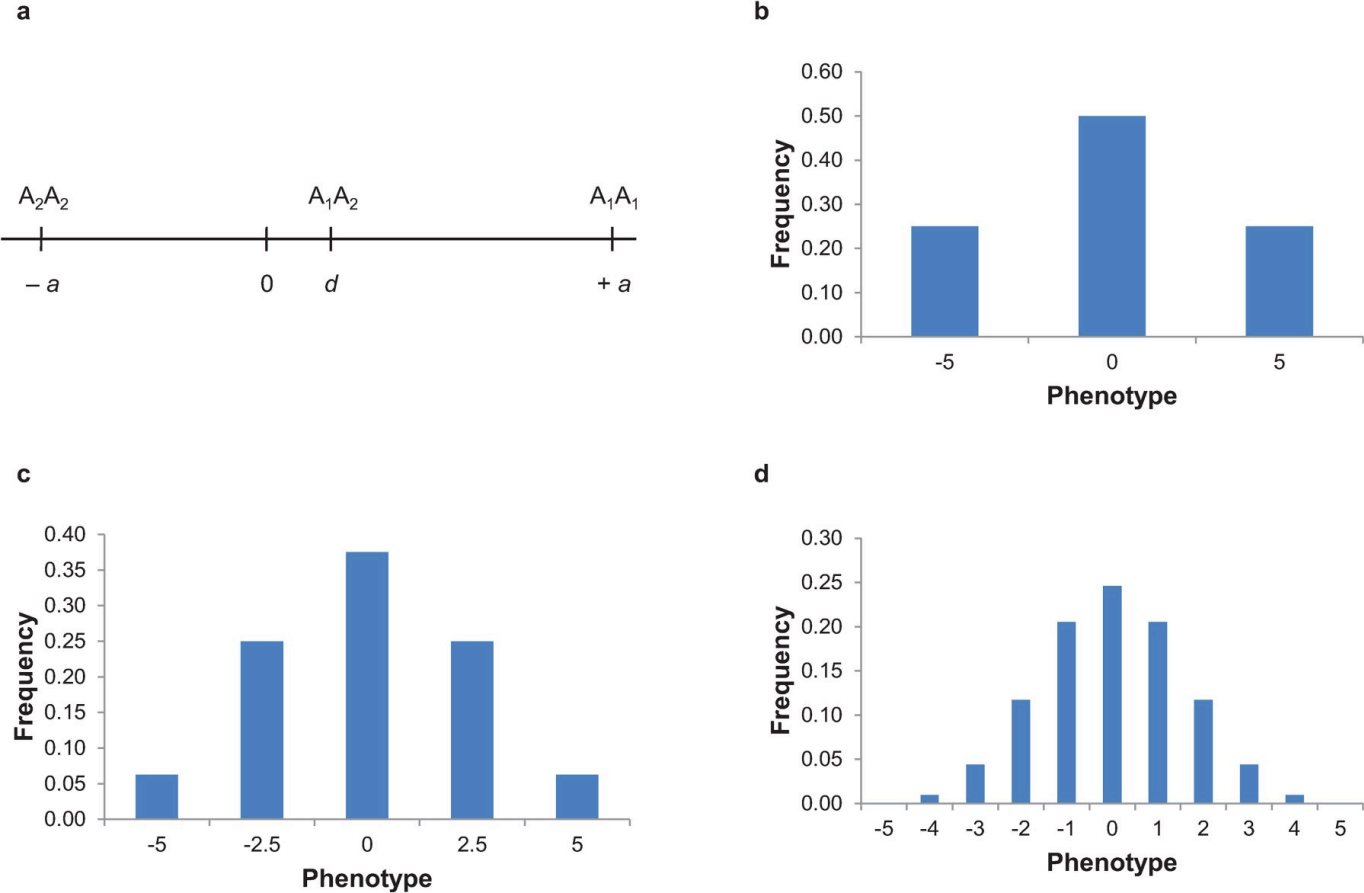
Fisher’s reconciliation of Mendel’s laws of inheritance with continuous variation for quantitative traits. (a) Fisher generalized Mendel’s phenotypes that have “a sharp and certain separation” [1] at a single locus for any phenotypic difference and any degree of dominance [4]. He centered the difference in phenotypes between the 2 homozygous genotypes at 0, so the genotype increasing the trait value has an effect of +*a* and the genotype decreasing the trait value has an effect of −*a*, where *a* is the additive effect. He defined the effect of the heterozygote, *d* (the dominance effect), as the difference between the average phenotype of heterozygotes and the average of the 2 homozygotes. (b) Fisher then assumed that each locus affecting a quantitative trait segregates in natural populations, with the frequencies *p* and *q* (*q* = 1–*p*) for the A_1_ and A_2_ alleles, respectively. The frequencies of the genotypes or phenotypes are given by the binomial expansion of (*p*+*q*)^*n*–1^, where *n* is the number of genotypes or phenotypes. With random mating, the frequencies for genotypes A_1_A_1_, A_1_A_2_, and A_2_A_2_ at each locus are *p*^2^, 2*pq*, and *q*^2^, respectively. In this example, *a* = 5, *d* = 0, and *p* = *q* = 0.5, so the 3 genotypes have phenotypes of 5, 0, and −5 with frequencies of 0.25, 0.5, and 0.25. These values of *p* and *q* correspond to the frequency of the 2 alleles in Mendel’s crosses of F_1_ hybrids, with the expected genotype ratios of 1:2:1 in the F_2_, but are generalized to any phenotypic effects and allele frequencies. (c) Fisher then assumed that as more loci affect the trait, the phenotypic range remains the same, and that the effects of all loci are the same or nearly so and add together to give the observed phenotype. Thus, as the number of loci increases, the effects of each on the phenotype become smaller. This example is for 2 loci (A and B), each with 2 alleles, with the same values of *a*, *d*, *p*, and *q* for each locus as that for locus A in panel (b). There are 9 genotypes but only 5 phenotypes. (d) The distinction between phenotypes decreases even further as the number of loci increases, and nongenetic environmental effects result in truly continuous distributions of quantitative traits in natural populations. In this example, there are 5 loci (A, B, C, D, and E), each with 2 alleles, with the same values of *a*, *d*, *p*, and *q* for each locus as that for locus A in panel (b). There are 243 genotypes but only 11 phenotypes.

Mendel’s principles of inheritance were contrary to the common observation at the time that crosses between organisms with different phenotypes were often intermediate between the 2 parents, and that phenotypic variation in populations is continuous and not discrete [2]. Indeed, Mendel deliberately excluded such phenotypes (or traits) from consideration. These continuously varying phenotypes, now recognized as quantitative traits, are more common in populations than phenotypes with the inheritance properties that Mendel reported. The relationships between relatives for quantitative traits are described in terms of regressions and correlations, as defined by the early biometricians Galton and Pearson [3]. It was not until 1918 that Ronald Fisher reconciled the 2 viewpoints [4] by showing that mendelian inheritance at a large (essentially infinite) number of loci would give rise to the observed continuous variation by generalizing Mendel’s principles to alleles with small effects, any type of dominance or epistasis, nongenetic (environmental) effects, and random mating populations (Fig 1). In the absence of knowledge of the individual genes and causal alleles, statistical models based on correlations of phenotypes between relatives are used to determine the fraction of variation for a quantitative trait in a population that is attributable to genetic variation, and to predict the response to selection.

Quantitative traits include all aspects of an organism’s morphology, physiology, behavior, and fitness, as well as molecular phenotypes such as gene expression, and protein and metabolite abundances. Although the principles of mapping regions of DNA associated with particular traits, known as quantitative trait loci (QTLs), by linkage to mendelian loci have been understood since the beginning of the 20th century [5], these studies were not widespread until the discovery of polymorphic molecular markers with strictly mendelian inheritance and the development of sophisticated statistical QTL mapping methods in the late 20th century [6]. Prior to that, mendelian and quantitative genetics were separate fields, with forward genetic screens for mendelian mutations in model organisms being used to determine the genetic basis of development, physiology and behavior, and quantitative genetic analysis of naturally occurring variation being used for genetic improvement of agricultural species and evolutionary biology.

What have we learned about the genetic basis of variation for quantitative traits from the thousands of studies mapping QTLs that have now been completed? First, quantitative traits are indeed highly polygenic, as suggested by Fisher [4]. Second, QTL effects estimated by averaging over different genetic backgrounds in the population are small, also consistent with Fisher’s hypothesis [4]. However, some observations hint that the true genetic basis of variation for quantitative traits is more complicated than there just being many loci with segregating alleles of small effects. When we introduce alleles associated with variation in a quantitative trait into common genetic backgrounds, we often find that the alleles have large effects that vary according to the genetic background [7]. Crossing mutations with large mendelian effects onto different naturally occurring genetic backgrounds in model organisms gives rise to a wide range of outcomes in which the mutant phenotype is exacerbated or ameliorated, revealing the existence of naturally segregating alleles that interact epistatically with the mutant allele. In humans, rare mendelian diseases typically have variable severity of symptoms, age of onset, and disease progression for different children with the same mutation, even within a family; again indicating the presence of naturally occurring epistatic modifiers. QTL mapping in model organisms has shown that QTL effects often depend on environmental context and sex [8]. Pervasive context-dependent effects mean that small effects of QTLs in populations may reflect averaging of true effects across multiple contexts.

These features of the genetic architecture of quantitative traits have implications for agriculture, human genetics, and evolution. For example, unless the relevant contexts are accounted for, attempts to improve traits of agricultural importance by transferring QTLs from breeds and varieties selected for performance under one set of conditions to breeds and varieties adapted to different conditions may not have the expected effects, and genomic prediction developed for one breed may not transfer to other breeds. Polygenic risk scores for human diseases that have been developed for one population may not be accurate in other populations unless specific interactions are included in the models. Identifying epistatic modifiers of rare human diseases could provide clues for therapies, and defining genotypes by their drug environment interactions will facilitate pharmacogenomic applications. Furthermore, context-dependent effects in natural populations may be in part responsible for the maintenance of quantitative genetic variation and adaptive evolution.

Mendel’s experiments on plant hybridization have laid the foundation of modern quantitative genetics. Mendel was well aware of the significance of his discoveries [9]. A few months before his death, during the investiture of his successor Franz Barina as abbot, he reportedly said “My scientific work has brought me great joy and satisfaction; and I am convinced that it won’t take long that the entire world will appreciate the results and meaning of my work” [10]. Although the accuracy of this quote cannot be unambiguously verified, Mendel would have been right. But little could he have predicted the enormous impact of his discoveries for agriculture, evolutionary biology, and precision medicine more than 150 years after publication of his treatise and 200 years after his birth.
